# Eating problems among old home care clients

**DOI:** 10.1002/cre2.585

**Published:** 2022-05-08

**Authors:** Annina Salmi, Kaija Komulainen, Annamari Nihtilä, Miia Tiihonen, Irma Nykänen, Sirpa Hartikainen, Anna L. Suominen

**Affiliations:** ^1^ Institute of Dentistry, School of Medicine, Faculty of Health Sciences University of Eastern Finland Kuopio Finland; ^2^ Social and Health Services City of Espoo Espoo Finland; ^3^ Kuopio Research Centre of Geriatric Care, School of Pharmacy, Faculty of Health Sciences University of Eastern Finland Kuopio Finland; ^4^ Institute of Public Health and Clinical Nutrition, Faculty of Health Sciences University of Eastern Finland Kuopio Finland; ^5^ Department of Oral and Maxillofacial Surgery University Hospital Kuopio Kuopio Finland

**Keywords:** chewing, eating, home care, older adults

## Abstract

**Aims:**

The purpose was to examine the prevalence and determinants of self‐reported eating problems in old home care clients, screened separately by a clinical nutritionist and a dental hygienist.

**Methods and Results:**

The data came from the Nutrition, Oral Health and Medication (NutOrMed) study, the participants of which were ≥75‐year‐old home care clients living in Finland. The structured interviews were conducted at the participants' (*n* = 250) homes. Of the participants, 29% reported poor appetite, 20% had problems with chewing, and 14% had problems with swallowing when asked by a clinical nutritionist. Additionally, 18% reported oral health‐related eating problems when asked by a dental hygienist. Participants with continuous xerostomia (odds ratio [OR]: 3.0, 95% confidence interval [CI]: 1.0–9.0) or poor self‐reported oral health (OR: 4.3, 95% CI: 1.4–13.0) had a higher risk for problems with chewing when asked by a clinical nutritionist. Edentulous participants (OR: 3.5, 95% CI: 1.2–10.9) and participants with toothache or problems with dentures (OR: 10.3, 95% CI: 4.0–26.0) had a higher risk for oral health‐related eating problems when asked by a dental hygienist.

**Conclusion:**

Eating problems are common in older adults, and interprofessional collaboration is required for their identification and alleviation.

## 1 INTRODUCTION

Decreased nutrient intake is often unidentified among vulnerable older adults and can consequently cause malnutrition (Elia et al., 2005; Kalyan, 2003). Malnutrition results from a long‐term lack of protein and/or energy. Malnutrition and the risk of malnutrition can not only be due to poor appetite or several diseases such as cognitive disorders but also due to medications, or financial or social factors. Malnutrition has many adverse effects on the older adult population, such as sarcopenia, a high risk for frailty as well as increased morbidity and mortality (McCormack, 1997; Saletti et al., 2005). In addition, malnutrition can impair the quality of life and can lead to increased healthcare costs and hospital stays (Lorefält et al., 2011; Tierney, 1996).

The determinants for deficiencies or an imbalance in nutritional status can be present in isolation or as a combination of a larger number of factors influencing dietary intake. Although older adults tend to have a decreased energy intake (de Groot et al., 2000), eating is much more than providing energy to the body. It is also the experiencing of a variety of foods and a part of the culture, and can therefore be considered an important matter. The enjoyability of mealtimes is associated with nutrition, subjective well‐being, and good health through the ability to maintain a varied diet (Iinuma et al., 2017). Oral health problems can negatively impact the eating of several food substances that require chewing, and can thus increase the risk of malnutrition (Fukutake et al., 2018; Gil‐Montoya et al., 2008; Kim & Jin, 2018; Lindroos et al., 2017; Quandt et al., 2010; Tada & Hiroko, 2014). Older adults usually have fewer natural teeth, which may cause difficulties in eating (Marcenes et al., 2003). People with fewer teeth tend to choose easy‐to‐chew foods and avoid hard‐to‐chew foods (Fukutake et al., 2018; Marcenes et al., 2003; Quandt et al., 2010; Walls et al., 2000). Tooth loss as well as a lower number of present teeth and occlusal contacts are associated with a lower intake of various foods and nutrients. Older adults with 20 or more natural teeth are more likely to have a normal body mass index (Marcenes et al., 2003; Nakamura et al., 2016). Dentures can help restore occlusal functions, but ill‐fitting dentures can lead to the avoidance of certain foods (Fukutake et al., 2018; Tada & Hiroko, 2014).

Our aim was to study and compare the prevalence of self‐reported eating problems and their determinants, as screened separately by a clinical nutritionist and a dental hygienist with slightly differently formatted questions.

## 2 MATERIALS AND METHODS

### 2.1 Design and participants

This study is part of the Nutrition, Oral Health and Medication (NutOrMed) study. The sample consisted of 75‐year‐old and older home care clients living in three cities in Eastern and Central Finland (Tiihonen et al., 2015), including a total sample of home care clients in one community and a randomized sample of home care clients in two other communities. A total of 440 participants were selected and were asked by home care nurses about their willingness to participate. If a participant was cognitively impaired, their proxy made the decision on participation. Written consents for the study were gathered from 300 home care clients or their proxies. After the gathering of consents, 4 participants had died, 3 had moved to another form of residence, and 43 refused to participate in the oral health structured interview and examination. Included in this study were those participants who answered the questions on eating problems and oral health‐related problems, asked by a clinical nutritionist and dental hygienist, respectively, with an eventual total of 250 participants. A more detailed description of the NutOrMed study protocol was published in 2015 (Tiihonen et al., 2015).

Homecare nurses, a clinical nutritionist, a pharmacist, and three dental hygienists conducted the structured interviews at the participants' homes. The nurses were the participants' own home care nurses, and the clinical nutritionist, the pharmacist, and the dental hygienist had previous experience in interviewing and assessing older persons. If a participant was not able to reply to the questions in the structured interview, a caregiver or nurse assisted the participant.

The two outcomes of this study were eating problems asked by a clinical nutritionist (related to chewing or swallowing, or poor appetite) and by a dental hygienist (related to oral health).

In the clinical nutritionist's structured interview, participants were asked about problems with chewing, swallowing, and poor appetite in the event that they had reported any difficulties with eating. The questions were “Do you have problems with chewing?,” “Do you have problems with swallowing?,” and “Do you have poor appetite?,” with the answering options “yes” or “no.” The clinical nutritionist also asked about a possible decrease in nutrition during the previous 3 months. The answering options were “significant,” “slight,” and “no change in nutrition.” In the analyses presented in Table 2, the first two options were combined and the answers were categorized as “decrease in nutrition” or “no changes.”

The nutritional screening was performed using the Mini Nutritional Assessment (MNA) tool. MNA is a standardized and validated nutritional status screening tool for older adults. Total scores of under 17 indicate malnutrition, scores of 17–23.5 a risk of malnutrition, and a score of over 23.5 normal nutrition (Guigoz et al., 2002).

A dental hygienist asked participants if they could maintain a varied diet without their gums or teeth causing problems, with three answer options provided: “no,” “occasionally,” or “continuously.” Those answering “no” or “occasionally” were considered as having problems with eating, and those answering “continuously” were considered as having no problems with eating. The dental hygienist also asked participants if they had toothache or other problems related to teeth or dentures during the previous 12 months, with the answering options of “no,” “occasionally,” and “continuously.” In the analyses, the answers “occasionally” and “continuously” were combined as “yes.” The dental hygienist also asked participants if they had xerostomia with the question “Do you have a feeling of dry mouth?.” The answering options were “no,” “occasionally,” and “continuously.” Participants' self‐reported oral health was classified as “good,” “poor,” or “doesn't know.” The dental hygienist also conducted a clinical examination with the participant sitting or lying down. As part of the clinical examination, the type and location of removable dental prostheses were registered. The previously published study protocol contains a detailed description of the clinical study (Tiihonen et al., 2015).

A home care nurse carried out the structured interview concerning sociodemographic factors, activities of daily living, instrumental activities of daily living, cognitive functioning, depressive symptoms, and health status. Functional ability was assessed by Instrumental Activities in Daily Living (IADL) with an 8‐item Lawton and Brody scale and scoring of 0‐8, with higher scores indicating better functioning. The Barthel Index was used to indicate dependence on a scale of 0 to 100, with lower scores indicating more severe dependence (Mahoney & Barthel, 1965). Comorbidity was defined using a modified version of the Functional Comorbidity Index (FCI). A higher sum score indicated greater comorbidity (Groll et al., 2005). Data on the following 13 medical conditions were used, and each condition was given one point: rheumatoid arthritis and other connective tissue inflammatory diseases, osteoporosis, chronic asthma or chronic obstructive pulmonary disease (COPD), coronary artery disease, myocardial infarction, heart failure, Parkinson's disease, stroke, diabetes mellitus, depressive disorder, visual impairment, hearing impairment, and dementing disease. Cognitive status was assessed with the Mini‐Mental State Examination (MMSE) on a scale from 0 to 30, where higher scores indicate better cognitive status (Folstein et al., 1975). A validated tool for the evaluation of depressive symptoms, a 15‐item geriatric depression scale (GDS‐15), was used for screening depressive symptoms, with higher scores indicating a potentially depressed individual (Yesavage & Sheikh, 1986). The participants' self‐reported health status was classified as “good,” “fairly good,” “moderate,” “fairly poor,” and “poor.” “Good” and “fairly good” were combined to simply “good,” and “fairly poor” and “poor” to simply “poor.” A nurse also asked about participants' self‐reported health, with the answering options “good,” “fairly good,” “moderate,” “fairly poor,” and “poor.” In the analyses in Table 2, the answers “good” and “fairly good” as well as “fairly poor” and “poor” were combined.

A pharmacist carried out the structured interview concerning the participants' drug use. All drugs, including prescriptions and over‐the‐counter drugs, as well as drugs taken regularly or as needed, were recorded. Information on drug use was collected on the basis of the structured interview (with assistance from nurses or family members), prescriptions, drug lists, packages, and dose dispensers. Regular drug use was categorized into three groups: 0–5 drugs, 6–9 drugs, and over 10 drugs in regular use (Viljakainen et al., 2016).

### 2.2 Statistical analysis

The data was analyzed using SPSS 27.0 software (Statistical Package for the Social Sciences), IBM Corporation, Armonk, New York, USA. Statistical comparisons for categorical variables were made using the *χ* ^2^ test and Mann–Whitney *U* or independent sample *t* test for continuous variables, considering *p* < .05 as significant. The results were reported as frequencies in percentages or means with standard deviations (SD). A binary logistic model was used to find adjusted associations between participants' oral health‐related characteristics and self‐reported problems in eating for age (continuous), gender, MMSE score, FCI score, 15‐item GDS‐15 score, MNA score, and the number of drugs in regular use.

### 2.3 Ethical approval

The study protocol was approved by the Research Ethics Committee of the Northern Savonia Hospital District, Kuopio, Finland. Written informed consent to participate in the study was provided by all participants or their proxy.

## 3 RESULTS

The mean age of participants was 84.5 years (SD 5.4), with a gender distribution of 74.0% females, and 64.8% of participants lived alone (Table 1). The proportion of edentate participants was 43.0%, of which 6.0% did not have any dentures. Of the participants, 27.6% had a combination of their own natural teeth and dental prostheses, and 29.2% had natural teeth and did not wear dentures. Comorbidities (mean FCI score 2.9, SD 1.9) and depressive symptoms (mean GDS‐15 score 4.8, SD 3.1) were common.

**Table 1:** Characteristics of the study participants (*n* = 250)

	*N* (%)	Mean (SD)
**Demographic characteristics**		
Age, mean (SD)		84.5 (5.4)
Female	185 (74)	
Living alone, missing *n* = 13	162 (64.8)	
Educational level in years		8.3 (3.5)
**Health‐related characteristics**		
IADL^a^ score, missing *n* = 7		4.6 (2.4)
Barthel Index^b^, mean (SD) missing *n* = 5		83.7 (18.8)
FCI score^c^, missing *n* = 2		2.9 (1.9)
MMSE^d^ score, missing *n* = 10		23.2 (5.3)
GDS‐15^e^, missing *n* = 5		4.8 (3.1)
Number of drugs in regular use, missing *n* = 3		
0–5 drugs	29 (11.6)	
6–9 drugs	80 (32.0)	
10 or more drugs	138 (55.2)	
Drugs in regular use, mean (SD), missing *n* = 3		10.3 (3.9)
Self‐reported health status, missing *n* = 2		
Good or fairly good	64 (25.6)	
Moderate	117 (46.8)	
Poor or fairly poor	67 (26.8)	
**Nutrition**		
Problems with chewing when asked by a clinical nutritionist	51 (20.4)	
Problems with swallowing when asked by a clinical nutritionist	34 (13.6)	
Poor appetite when asked by a clinical nutritionist	72 (28.8)	
Mini Nutritional Assessment score^f^		21.9 (2.6)
Nutrition by Mini Nutritional Assessment score		
Normal nutrition	37 (14.8)	
Malnutrition or risk of malnutrition	213 (85.2)	
Decrease in food intake in the past 3 months	54 (22.4)	
**Oral health‐related characteristics**		
Problems with eating when asked by a dental hygienist	46 (18.4)	
Xerostomia		
Occasional	109 (43.6)	
Continuous	31 (12.4)	
Denture status		
Dentate, no removable dentures	73 (29.2)	
Dentate, removable dentures	69 (27.6)	
Edentulous, no removable dentures or fully removable dentures	108 (43.2)	
Self‐reported oral health status		
Good	173 (69.2)	
Poor	35 (14.0)	
Doesn't know	42 (16.8)	
Toothache or problems with dentures	72 (28.8)	

^a^ IADL Instrumental Activities in Daily Living scoring 0‐8, with higher scores indicating better functioning.

^b^ Barthel Index scale from 0 to 100, lower scores indicating more severe dependence.

^c^ FCI Functional comorbidity, higher sum score represents greater comorbidity.

^d^ MMSE Mini‐Mental State Examination, range 0–30, higher scores indicate better functioning.

^e^ GDS‐15 15‐item Geriatric Depression Scale, range 0–15, higher scores (>6) indicate a potentially depressed individual.

^f^ MNA Mini Nutritional Assessment score, range 0–30, scores 24–30 indicate normal nutrition, scores less than 17 indicate malnutrition.

When asked by a clinical nutritionist, 20.4% had problems with chewing (Table 1), 13.6% with swallowing, and 28.8% had a poor appetite. When asked by a dental hygienist, 18.4% of the participants responded that they had oral health‐related eating problems.

In binary analyses (Table 2), factors associated with problems with chewing, when asked by a clinical nutritionist, were participants' poor self‐reported general health status (*p* = .045), the risk of malnutrition (*p* = .044), a decrease in food intake in the past 3 months (*p* = .001), and poor self‐reported oral health status (*p* = .002). Factors associated with problems with swallowing, when asked by a clinical nutritionist, were participants' depressive symptoms (indicated by higher GDS‐15 scores) (*p* = .012), the risk of malnutrition (*p* = .004), and poor self‐reported oral health status (*p* = .030). Factors associated with participants' poor appetite, when asked by a clinical nutritionist, were participants' depressive symptoms (indicated by a higher GDS‐15 score) (*p* = .009), a high number of drugs in regular use (*p* < .001), poor self‐reported health status (*p* = .048), a lower MNA score (*p* < .001), malnutrition or a risk of malnutrition according to the MNA score (*p* = .026), a decrease in food intake in the past 3 months (*p* < .001), and the incidence of xerostomia (*p* = .020).

**Table 2:** Reported problems with eating by eating‐related characteristics and nutrition

	Asked by a clinical nutritionist						Asked by a dental hygienist	
	Problems with chewing		Problems with swallowing		Poor appetite		Oral health‐related eating problems	
		*p* Value*		*p* Value*		*p* Value*		*p* Value*
**Demographic characteristics**								
Age, mean (SD)	84.2 (5.6)	0.567	83.0 (5.9)	0.102	84.7 (5.3)	0.539	83.5 (5.4)	0.079
Female *n* (%)	36 (19.5)	0.534	23 (12.4)	0.364	57 (30.8)	0.236	29 (15.7)	0.061
Living alone *n* (%)	34 (21.0)	0.764	23 (14.2)	0.571	45 (27.8)	0.277	24 (14.8)	0.127
Educational level in years, mean (SD)	7.8 (3.4)	0.380	8.3 (4.1)	0.592	8.0 (2.9)	0.972	8.4 (3.5)	0.487
**Health‐related characteristics**								
IADL^a^ mean (SD)	4.6 (2.6)	0.971	4.5 (2.8)	0.938	4.5 (2.4)	0.478	4.8 (2.4)	0.537
Barthel Index^b^, mean (SD)	79.7 (21.9)	0.108	77.1 (26.1)	0.123	83.2 (19.5)	0.933	84.0 (13.7)	0.217
FCI^c^ mean (SD)	2.8 (1.9)	0.618	3.2 (2.2)	0.343	3.1 (1.8)	0.241	3.6 (2.0)	0.013
MMSE^d^ mean (SD)	23.1 (6.4)	0.464	23.0 (7.3)	0.357	22.8 (4.8)	0.218	23.2 (4.8)	0.882
GDS‐15^e^ mean (SD)	5.6 (3.8)	0.188	6.3 (3.8)	0.012	5.7 (3.5)	0.009	6.3 (3.7)	0.002
Number of drugs in regular use, *n* (%)		0.984		0.264		0.000		0.038
0–9 drugs	22 (20.6)		12 (11.0)		18 (16.5)		14 (12.8)	
10 or more drugs	28 (20.3)		22 (15.9)		54 (39.1)		32 (23.2)	
Drugs in regular use, mean (SD)	10.5 (4.1)	0.536	10.8 (4.3)	0.342	11.5 (3.3)	0.000	12.0 (4.3)	0.002
Self‐reported health status, *n* (%)		0.045		0.275		0.048		0.000
Good or fairly good	13 (20.3)		8 (12.5)		14 (21.9)		4 (6.3)	
Moderate	17 (14.5)		13 (11.1)		31 (26.5)		19 (16.2)	
Poor or fairly poor	20 (29.9)		13 (19.4)		27 (40.3)		22 (32.8)	
**Nutrition**								
Mini Nutritional Assessment (MNA)^f^, mean (SD)	21.2 (2.8)	0.044	20.5 (3.2)	0.004	21.0 (2.5)	0.000	20.8 (2.3)	0.000
Nutrition according to MNA score *n*, (%)		0.494		0.592		0.026		0.080
Malnutrition or risk of malnutrition	45 (21.1)		30 (14.1)		67 (31.5)		43 (20.2)	
Normal nutrition	6 (16.2)		4 (10.8)		5 (13.5)		3 (8.1)	
Decrease in food intake in the past 3 months	20 (35.7)	0.001	12 (21.4)	0.052	34 (60.7)	0.000	16 (28.6)	0.026
**Oral health‐related characteristics**								
Xerostomia		0.078		0.712		0.020		0.058
Occasional	21 (19.3)		17 (15.6)		32 (29.4)		21 (19.3)	
Continuous	11 (35.5)		4 (12.9)		15 (48.8)		10 (32.3)	
Denture status		0.111		0.320		0.098		0.240
Dentate, no dentures	14 (19.2)		10 (13.7)		20 (27.4)		9 (12.3)	
Dentate, removable dentures	9 (13.0)		6 (8.7)		14 (20.3)		13 (18.8)	
Edentulous, no dentures or full dentures	28 (25.9)		18 (16.7)		38 (35.2)		24 (22.2)	
Self‐reported oral health status		0.002		0.030		0.291		0.002
Good	25 (14.5)		17 (9.8)		48 (27.7)		23 (13.3)	
Poor	13 (37.1)		7 (20.0)		8 (22.9)		13 (37.1)	
Doesn't know	13 (31.0)		10 (23.8)		16 (38.1)		10 (23.8)	
Toothache or problems with dentures	14 (21.2)	0.068	11 (15.3)	0.623	12 (18.2)	0.278	31 (43.1)	0.000

^a^ IADL Instrumental Activities in Daily Living scoring 0‐8, with higher scores indicating better functioning.

^b^ Barthel Index scale from 0 to 100, lower scores indicating more severe dependence.

^c^ FCI Functional comorbidity, a higher sum score represents greater comorbidity.

^d^ MMSE Mini‐Mental State Examination, range 0–30, higher scores indicate better functioning.

^e^ GDS‐15 15‐item Geriatric Depression Scale, range 0–15, higher scores (>6) indicate a potentially depressed individual.

^f^ MNA Mini Nutritional Assessment score, range 0–30, scores 24–30 indicate normal nutrition, scores less than 17 indicate malnutrition.

* *χ* ^2^ and Mann–Whitney *U* or independent sample *t* test for continuous variables, considering *p* < .05 as significant.

Furthermore, in binary analyses, the factors associated with oral health‐related eating problems, when asked by a dental hygienist, were participants' high comorbidity according to FCI (*p* = .013), depressive symptoms (indicated by a higher GDS‐15 score) (*p* = .002), a high number of drugs in regular use (*p* = .002), poor self‐reported health status (*p* < .001), a risk of malnutrition (lower MNA score) (*p* < .001), a decrease in food intake in the past 3 months (*p* = .026), poor self‐reported oral health status (*p* = .002), and toothache or problems with dentures (*p* < .001).

Based on logistic regression analysis (Table 3), those who had continuous xerostomia (odds ratio [OR]: 3.0, 95% confidence interval: 95% CI: 1.0–9.1) or poor self‐reported oral health (OR: 4.3, 95% CI: 1.4–13.1) had a higher risk of problems with chewing when asked by a clinical nutritionist. Edentulous participants (OR: 3.5, 95% CI: 1.2–10.9) and participants with toothache or problems with dentures (OR: 10.3, 95% CI: 4.0–26.0) had a higher risk of oral health‐related eating problems when asked by a dental hygienist.

**Table 3:** Adjusted^a^ associations between participants' (*n* = 235) oral health‐related characteristics and self‐reported problems with eating from multivariate logistic regression (95% CI, 95% confidence interval; OR, odds ratio)

	Asked by a clinical nutritionist									Asked by a dental hygienist		
	Problems with chewing			Problems with swallowing			Poor appetite			Oral health‐related eating problems		
	OR	95% CI	*p* Value	OR	95% CI	*p* Value	OR	95% CI	*p* Value	OR	95% CI	*p* Value
Denture status (ref. dentate, no dentures)			0.013			0.173			0.170			0.068
Dentate with dentures	0.48	0.20–1.41	0.182	0.47	0.14–1.64	0.239	0.63	0.26–1.56	0.320	1.59	0.46–5.50	0.466
Edentulous^b^	2.06	0.86–4.86	0.098	1.43	0.54–3.74	0.477	1.38	0.65–2.94	0.405	3.51	1.23–10.88	0.030
Xerostomia (ref. absence of xerostomia)			0.078			0.747			0.137			0.530
Occasional xerostomia	0.97	0.42–2.25	0.935	1.35	0.53–3.45	0.535	1.19	0.57–2.48	0.638	1.61	0.61–4.25	0.335
Continuous xerostomia	3.01	1.01–9.06	0.049	0.91	0.22–3.79	0.895	2.66	0.99–7.15	0.052	1.89	0.53–6.75	0.328
Self‐reported oral health (ref. good)			0.017			0.382			0.159			0.996
Poor	4.34	1.44–13.07	0.009	1.12	0.31–4.1	0.851	0.50	0.17–1.50	0.218	0.96	0.92–3.12	0.939
Doesn't know	2.74	1.08–6.98	0.035	2.01	0.72–5.81	0.177	1.59	0.69–3.68	0.278	1.01	0.318–3.22	0.984
Occurrence of tooth‐ache/problems with dentures (ref. no)^c^	1.00	0.4–2.3	0.98	0.96	0.36–2.56	0.928	1.02	0.49–2.14	0.956	10.321	4.01–26.02	<0.001

^a^ For age (continuous), gender, Mini‐Mental State Examination score (MMSE^d^) score, Functional comorbidity (FCI)^e^ score. 15‐item Geriatric Depression Scale (GDS‐15)^f^ score, Mini Nutritional Assessment (MNA)^g^ score, number of drugs in regular use.

^b^ Includes edentulous with and without dentures.

^c^ Occasional or continuous.

^d^ MMSE score, range 0–30, higher scores indicate better functioning.

^e^ Functional comorbidity (FCI) score, a higher sum score represents greater comorbidity.

^f^ 15‐item Geriatric Depression Scale (GDS‐15) score, range 0–15, higher scores (>6) indicate a potentially depressed individual.

^g^ Mini Nutritional Assessment (MNA) score, range 0–30, scores 24–30 indicate normal nutrition, scores less than 17 indicate malnutrition.

Furthermore, participants' better nutrition (higher MNA score) (OR: 0.8, 95% CI: 0.7–0.9) was associated with a lower risk of problems with swallowing (OR: 0.78, 95% CI: 0.6–0.9) and a lower risk of poor appetite (OR: 0.80, 95% CI: 0.7–0.9), when asked by a clinical nutritionist (data not shown). A higher number of drugs in regular use was associated with a higher risk of poor appetite (OR: 1.1, 95% CI: 1.1–1.2) and oral health‐related eating problems (OR: 1.2, 95% CI: 1.0–1.3), both when asked by a clinical nutritionist.

## 4 DISCUSSION

Eating‐related problems were common in old home care clients. When asked by a clinical nutritionist, nearly one‐third had a poor appetite, one‐fifth had problems with chewing and one in seven had problems with swallowing. When asked by a dental hygienist, one‐fifth responded that they had oral health‐related eating problems. All reported eating problems were associated with the participants' poorer nutritional state and decreased food intake during the past 3 months. Participants who reported poor oral health status had more problems related to chewing and swallowing. Oral health‐related eating problems were more common in edentulous participants and in those who had toothache or problems with dentures. In addition, participants with xerostomia reported more eating problems than those without dry mouth problems.

Poor appetite was the most common eating‐related finding in our study, with almost one‐third of participants reporting a poor appetite. The proportion of poor appetite was slightly higher than that reported by van der Meij et al. (2017), which may be explained by the more vulnerable group of community‐dwelling older adults included in our study. Van der Meij et al. (2017) found that community‐dwelling older adults aged between 70 and 79 in the United States with poor appetite had different and suboptimal eating patterns compared to older adults with a good appetite, and their intake of crucial nutrients was diminished. According to the findings of Van der Meij and the review article of Fávaro‐Moreira et al. (2016), poor appetite is a significant risk factor for malnutrition. In our study, we found that decreased food intake in the past 3 months was associated with a poor appetite. Sieske et al. (2019) reported a significant association between inflammation and a decreased appetite with decreased food intake among geriatric patients in acute hospital care in Germany. Studies suggest the recognition of poor appetite and treatment of the underlying cause, as poor appetite has a significant worsening impact on the nutritional status of older adults.

In our study, poor self‐reported oral health status was associated with problems with chewing and swallowing. This supports previous findings suggesting that oral health status, as measured by the ability to chew, has a direct effect on the swallowing function (Furuta et al., 2013). Severe cognitional impairment can disrupt denture‐wearing, resulting in problems with chewing and swallowing and may possibly affect nutrition (Furuta et al., 2013). Furthermore, Nakanishi et al. (1999) found that chewing problems were associated with a greater likelihood of poor general health and decreased quality of life in older adults in Japan. In our study, poor self‐reported general health was associated with chewing problems, which supports the findings of the Japanese study (Nakanishi et al., 1999). On the other hand, some studies have suggested that dietary selection and nutritional state are influenced by age, socioeconomic status, and general health rather than solely by issues concerning oral health (Allen & McMillan, 2003). In our study, a higher GDS‐15 score indicating depressive symptoms was associated with problems with swallowing and poor appetite. However, the score was interpreted as a continuous variable, and it does not take into account the cut‐off value usually used for identifying potentially depressed individuals. Depressive symptoms or other factors contributing to poor general health may also cause neglect of cooking and eating. Older adults who live alone may feel lonely and their eating routines may change. General health and cognitive status should be noted together with oral health as factors contributing to nutritional status.

The role of occlusal functioning is also evident in our study, as edentate participants (with or without dentures) had more oral health‐related eating problems. This finding is in line with a Finnish population‐based study of food consumption and nutrient intake related to denture use in an older age group (Jauhiainen et al., 2017). In our study, participants with a combination of natural teeth and prostheses tended to have a lower risk of chewing or swallowing problems or poor appetite. This finding corresponds to that of Azzolino et al. (2019), and the better occlusal functioning resulting from the combination of own natural teeth and dentures can be explained by the low number of natural teeth of our old and vulnerable participants. The lack of posterior occlusal teeth pairs decreases the ability to eat healthy food, and edentate individuals have a lower calorie intake (Sahyoun et al., 2003). In our study, edentate participants had a higher risk of eating problems. Our findings are in agreement with another Finnish study, which found an association between edentulousness and malnutrition according to MNA (Saarela et al., 2014). Especially in older adults, the loss of natural teeth is related to a diminished nutritional intake (Jauhiainen et al., 2017). Poor self‐reported oral health increases frailty (Shwe et al., 2019). Compared to problems with chewing, the lower reported proportion of oral health‐related eating problems (as questioned by the dental hygienist) may also be explained by participants' fear of dental procedures and being nervous about visiting a dentist. Adaptation and fear of dental procedures could cause an underrating of oral health‐related eating problems. Dental fear is one of the major reasons for the avoidance of dentists (Beaudette et al., 2017).

Participants with xerostomia had more problems with chewing and more often a poor appetite compared to those without dry mouth problems, which is in line with the results of Barbe (2018). In our study, over two in five reported occasional and one in eight continuous xerostomia. Many of the participants in this study had polypharmacy (over 10 drugs in regular use), which can increase the risk of xerostomia, especially as it can be associated with many common drug groups prescribed to old people (Barbe, 2018). A higher number of drugs was associated with participants' poor appetite and oral health‐related eating problems, which is in line with the results of Nakamura et al. (2021).

The strengths of this study were its multidisciplinary approach, a high number of validated instruments (nutrition, morbidity, cognition), and a population‐based design. The questions on participants' eating problems were asked by both a clinical nutritionist and a dental hygienist and as far as we know, other similar studies do not exist. Studies of noninstitutionalized older adults, in which the oldest and the most vulnerable individuals are also included, are scarce. In our study, participants lived at home and received home care. No one was excluded based on their age, morbidity, cognitive impairment, or other factors. Data were collected through structured interviews in person by trained professionals who had previous experience in working with older adults. Every participant had their proxy or personal nurse who knew the patient answering the questions, which was likely to increase the probability of the participants understanding the questions and the reliability of their answers. Information on drug use was collected with the help of nursing staff or family members from prescriptions, drug lists, packages, and dose dispensers. For nutritional status, we used an MNA test validated and developed for older adults, which also supports the validity of the data.

One limitation of the study was its cross‐sectional design, whereby causality could be determined. Another limitation was the relatively small sample size, shown by the small number of participants in some subgroups. A possible limitation was also the fact that the clinical nutritionist presented simple and straightforward questions about the participants' eating problems, whereas the corresponding questions asked by the dental hygienist may have been more complex and thus more challenging to understand. However, all the questions were pilot tested with a small number of older adults (*n* = 8) and were found useful and easy to understand. No requirement to revise questions measuring eating problems was evident. It is also known that older adults underestimate their oral health problems and adapt to them, and our participants may have responded accordingly. For example, it has been found that older adults who have been edentulous for over 10 years are more likely to accept the limitations of full dentures and report fewer problems than adults who have been edentulous for less than 10 years (Allen & McMillan, 2003). Furthermore, given the participants with impaired cognition, the relatively long 12‐month reporting period used for self‐evaluation of toothache or problems with dentures could be considered a limitation of this study. However, a proxy or the own caregiver of a participant was included in the interview to minimize the effect of this limitation. Furthermore, a series of questions about feelings and symptoms of dry mouth could have provided more information on xerostomia in this group of participants. Xerostomia is a self‐reported feeling of dry mouth, which in this study was assessed with the question “Does your mouth feel dry?.” One of the reasons why we chose to ask a simple and straightforward question instead of presenting a larger questionnaire on a specific subject was because the structured interviews by nutritionists and dental hygienists were time‐consuming and potentially strenuous for this vulnerable group of participants.

In this study, we focused on problems with eating, and some factors contributing to eating problems were identified. Our findings on the association between poor appetite and malnutrition as well as edentulousness and malnutrition correspond to those in a contemporary review (Ástvaldsdóttir et al., 2018). As Azzolino et al. (2019) previously published, our study also found that participants' risk of malnutrition was associated with problems with chewing, swallowing, and poor appetite as well as with oral health‐related problems with eating. The problems with eating are multifactored, and an adaptation to impaired conditions and the framework of other people of the same age can cause the underrating of difficulties in eating and of oral health‐related problems. It also appears that answers may vary depending on who is asking, how the question is formulated, and the context. Malnutrition is a multifactored problem that should be prevented and treated holistically (Toniazzo et al., 2017).

## 5 CONCLUSION

Good oral health is important for the ability to eat in older adults. Poor appetite was the most common finding, indicating that oral health‐related problems are only one part of a wider range of eating problems. As the problems in eating are multifactored, the collection of information on eating problems should be broad, continuous, and multiprofessional. Interprofessional collaboration is required to identify and alleviate eating problems, as answers may vary depending on which healthcare professional is asking.

## AUTHOR CONTRIBUTIONS

Annina Salmi wrote the manuscript with the support of Kaija Komulainen, Annamari Nihtilä, Miia Tiihonen, Irma Nykänen, Sirpa Hartikainen, and Anna L. Suominen. Irma Nykänen, Miia Tiihonen, Sirpa Hartikainen, and Anna L. Suominen have participated in designing the Nutormed study. Irma Nykänen and Miia Tiihonen have participated in collecting the data. Annina Salmi has analyzed the data with the support of Kaija Komulainen, Annamari Nihtilä, and Anna L. Suominen. All authors discussed the results and contributed to the final manuscript.

## CONFLICT OF INTEREST

The author declares no conflict of interest.

## Data Availability

The data sets generated and/or analyzed during the current study are not publicly available due to ethical requirements by the ethics committee.
